# Effects of dihydroorotate dehydrogenase inhibition and vitamin E supplementation on ferroptosis-related mechanisms and post-mortem meat quality in broiler chickens

**DOI:** 10.5713/ab.250847

**Published:** 2026-03-11

**Authors:** Tomonori Nakanishi, Saki Nemoto, Laurie Erickson, Satoshi Kawahara

**Affiliations:** 1 Department of Biochemistry and Applied Biosciences, Faculty of Agriculture, University of Miyazaki, Miyazaki, Japan; 2 Unit of Animal and Plant Biosciences, Faculty of Agriculture, University of Miyazaki, Miyazaki, Japan; 3 Department of Animal and Grassland Sciences, Faculty of Agriculture, University of Miyazaki, Miyazaki, Japan; 4 Retired from Sarah Hartman Women’s College of Touro, Chicago, IL, USA

**Keywords:** Broiler Chicken, Dihydroorotate Dehydrogenase, Ferroptosis, Meat Quality, Vitamin E

## Abstract

**Objective:**

This study investigated the role of ferroptosis, an iron-dependent form of regulated cell death, in meat quality by comparing the effects of pre-slaughter administration of teriflunomide, an inhibitor of dihydroorotate dehydrogenase (DHODH), which can induce ferroptosis, and dietary vitamin E, an antioxidant that can suppress ferroptosis, on the post-mortem biochemical properties of broiler chicken muscle.

**Methods:**

Broiler chickens were randomly allocated to three groups: control, teriflunomide-treated, and vitamin E-supplemented. Teriflunomide was administered subcutaneously prior to slaughter, and vitamin E was provided in the diet. Post-mortem thigh muscles were analyzed for oxidative status, such as lipid peroxide levels and the ratio of reduced to oxidized glutathione (GSH/GSSG); the expression of ferroptosis-related genes, such as glutathione peroxidase 4 (*GPX4*) and acyl-CoA synthetase long-chain family member 4 (*ACSL4*); and meat quality traits such as pH, color, and water-holding capacity.

**Results:**

Teriflunomide significantly reduced the GSH/GSSG ratio (p<0.001) and increased *ACSL4* expression compared with the control and vitamin E groups (p<0.05), indicating elevated oxidative stress. Vitamin E significantly increased *GPX4* expression compared with teriflunomide (p<0.05). Lipid peroxidation was numerically lower in the vitamin E group than in the other groups, although the differences were not statistically significant (p = 0.062). Teriflunomide slowed post-mortem pH decline (p<0.001) and decreased L* and b* values, whereas a* values were higher (p<0.05). Vitamin E maintained higher L* and b* values and slightly lower a* values than the control (p<0.05). No statistically significant differences were observed in water-holding capacity among the groups.

**Conclusion:**

These findings indicate that ferroptosis-related processes play a critical role in regulating post-mortem meat quality in broiler thigh muscles. DHODH inhibition by teriflunomide and antioxidant treatment with vitamin E yielded contrasting effects.

## INTRODUCTION

Meat quality is one of the most important factors determining consumer preference and economic value in meat production, and its improvement remains a major goal in animal food research. Meat quality is influenced by characteristics such as color, water-holding capacity, tenderness, and flavor, all of which are closely related to the structural and biochemical properties of muscle tissue [1]. Among the factors affecting meat quality, biochemical changes that occur before and after slaughter, including alterations in energy metabolism, redox balance, and the progression of cell death, play a crucial role in the conversion of muscle into meat during post-mortem aging [2,3]. However, the molecular mechanisms underlying these biochemical events and their contribution to meat quality have not yet been fully clarified. A deeper understanding of these processes is essential for the development of new approaches to improve and control meat quality.

Ferroptosis is a form of regulated cell death characterized by the iron-dependent accumulation of lipid peroxides within biological membranes, which distinguishes it from apoptosis and necrosis [4]. Unlike apoptosis, ferroptosis does not involve caspase activation or typical nuclear fragmentation [4]. While it ultimately results in membrane rupture, this occurs as a regulated consequence of lipid peroxidation rather than sudden, uncontrolled necrotic lysis [4]. This process is tightly regulated by glutathione peroxidase 4 (GPX4) and has been implicated in various physiological and pathological conditions [5]. Recent studies have suggested a potential link between ferroptosis and meat quality traits. Ferroptosis-related oxidative damage has been reported to influence the water-holding capacity of beef [6]. Our previous study using chickens also demonstrated that the pre-slaughter activity of GPX4 affected post-mortem meat quality, including pH and color parameters [7]. These observations highlight the involvement of ferroptosis in the biochemical processes underlying meat quality and suggest that further investigation may provide new insights into the post-mortem conversion of muscle to meat.

Dihydroorotate dehydrogenase (DHODH) is a mitochondrial enzyme that catalyzes a key step in *de novo* pyrimidine biosynthesis, facilitating the conversion of dihydroorotate to orotate, and thereby contributing to nucleotide production essential for cellular proliferation and metabolism [8]. Beyond its classical role in pyrimidine metabolism, DHODH has recently been recognized as a crucial regulator of ferroptosis. Specifically, DHODH functions as an alternative antioxidant system within mitochondria, where it reduces ubiquinone to ubiquinol, mitigating lipid peroxidation and suppressing ferroptotic cell death independently of GPX4 [9]. Inhibition of DHODH has been shown to sensitize cells to ferroptosis, highlighting its importance in maintaining mitochondrial integrity under oxidative stress [10]. Despite these advances in understanding the biochemical functions of DHODH, the potential relationship between DHODH activity and post-mortem meat quality remains completely unexplored.

Vitamin E is a lipid-soluble antioxidant that has long been used as a dietary supplement in livestock production to improve meat quality. Numerous studies have demonstrated that dietary supplementation with vitamin E reduces lipid oxidation in muscle tissues, thereby improving color stability, water-holding capacity, and oxidative stability during storage [11]. These effects are mainly attributed to the ability of α-tocopherol to scavenge lipid radicals and terminate lipid peroxidation chain reactions [12]. While its antioxidant properties are well established, recent studies using cultured mouse hematopoietic stem and progenitor cells have shown that vitamin E can inhibit ferroptosis by preventing lipid peroxide accumulation [13]. Although vitamin E does not directly interact with ferroptosis-regulating enzymes such as GPX4 or DHODH, it can act in parallel with these endogenous systems to protect cellular membranes from oxidative damage [14]. However, the involvement of ferroptosis-related pathways in the effects of dietary vitamin E on post-mortem meat quality has rarely been investigated.

To further explore the relationship between ferroptosis and meat quality, the present study was designed to investigate broiler chickens receiving teriflunomide, a DHODH inhibitor, or given dietary vitamin E prior to slaughter, alongside an untreated control group. Teriflunomide treatment was intended to promote ferroptosis, while vitamin E supplementation was expected to suppress it. Comparison of post-mortem muscle biochemical properties among these groups was performed to gain deeper insight into the role of ferroptosis in meat quality.

## MATERIALS AND METHODS

### Animals and treatments

One-week-old male chicks (Ross strain) were obtained from a local hatchery and housed in the animal facility of the University of Miyazaki under controlled conditions (28±2°C). During the one-week rearing period, the chicks had free access to commercial feed (JA Miyazaki-chuo) and tap water. At 14 days of age, the animals were randomly assigned to three groups (n = 6 per group): control, teriflunomide-treated, and vitamin E-supplemented. The vitamin E group received a diet supplemented with 500 mg α-tocopherol/kg feed for one week, whereas the other two groups were given the same basal diet without supplementation. The teriflunomide group was administered teriflunomide, a DHODH inhibitor, dissolved in 25% dimethylformamide and 75% propylene glycol, by subcutaneous injection at a dose of 5 mg/kg body weight at 48, 24, and 2 h before euthanasia. The control and vitamin E groups received vehicle injections only. The broiler chickens were reared on the floor as a single group in a 140 cm×170 cm pen until 14 days of age, prior to group allocation. After grouping, the birds were housed individually in 30 cm×60 cm cages for one week, during which body weight and feed intake were recorded daily. Each individual bird was considered as an experimental unit. At 21 days of age, all animals were euthanized under isoflurane inhalation anesthesia, and both left and right thigh muscles were collected. The muscle samples were placed in a food-grade polypropylene container with a lid (10×7×4.5 cm) and stored at 4°C for 72 h.

To investigate the effects of each experimental treatment on post-mortem changes in chicken meat, we analyzed thigh muscles not only immediately after slaughter but also at 72 h post-mortem, focusing on parameters related to ferroptosis (glutathione content and lipid peroxide levels) as well as meat quality traits such as pH, color, and water-holding capacity. At 72 h post-mortem, biochemical changes such as pH decline, rigor mortis, and its resolution are generally completed, resulting in relatively stable meat quality [15]. Glutathione content and lipid peroxide levels were assessed at both 0 and 72 h post-mortem, while color and pH were monitored repeatedly during this period. Water-holding capacity was evaluated at 72 h post-mortem based on drip loss during refrigerated storage and thawing loss after a freeze-thaw cycle. In contrast, gene expression analysis was performed only immediately after slaughter, as total RNA could not be reliably extracted from muscles at 72 h post-mortem.

### Measurement of glutathione concentration

Approximately 100 mg of the right thigh muscle (iliotibialis lateralis) was collected immediately after slaughter and after 72 h of refrigerated storage, and samples were stored at −80°C until analysis. Glutathione levels were determined using a commercial glutathione assay kit (Dojindo Laboratories) according to the manufacturer’s instructions. The ratio of reduced glutathione (GSH) to oxidized glutathione (GSSG) was calculated for each sample.

### Determination of lipid peroxides

For the assay of lipid peroxides, 500 mg of the right thigh muscle (iliotibialis lateralis) was excised immediately after slaughter and after 72 h of refrigerated storage. The samples were stored at −80°C until analysis and processed according to a previously reported method [7]. Briefly, muscle tissues were homogenized, deproteinized with sodium tungstate and sulfuric acid, and reacted with 1,3-diethyl-2-thiobarbituric acid (DETBA). The DETBA-malondialdehyde (MDA) adducts were separated using high-performance liquid chromatography equipped with an Inertsil ODS-3 column (5 μm, 4.6×250 mm; GL Sciences) and detected with a fluorescence detector (excitation wavelength: 515 nm; emission wavelength: 555 nm). A calibration curve was constructed using 1,1,3,3-tetraethoxypropane as an external standard, and the lipid peroxide concentration in muscle samples was determined by interpolation.

### Expression analysis of ferroptosis-related genes

Right thigh muscle (iliotibialis lateralis) samples weighing approximately 50 mg were collected immediately after slaughter and stored at −80°C until analysis. Total RNA was extracted using Isogen (Nippon Gene), and 500 ng of RNA were reverse-transcribed into cDNA with ReverTra Ace (TOYOBO). Quantitative reverse transcription-polymerase chain reaction was performed on the AriaMx Real-Time PCR system (Agilent Technologies) using the Brilliant III Ultra-Fast SYBR Green QPCR Master Mix (Agilent Technologies) according to the manufacturer’s instructions. The transcription levels of four ferroptosis-related genes—*DHODH*, *GPX4*, ferroptosis suppressor protein 1 (*FSP1*), and acyl-CoA synthetase long-chain family member 4 (*ACSL4*)—were quantified using primer sequences shown in Table 1. Glyceraldehyde-3-phosphate dehydrogenase (*GAPDH*) served as the reference gene. Cycle threshold values were determined during the exponential phase of amplification, and specificity of the PCR products was confirmed by melting curve analysis. Relative expression levels of ferroptosis-related genes were normalized to *GAPDH*.

### Measurements of pH and color

The right thigh muscles (iliotibialis lateralis) were subjected to pH and color measurements during refrigerated storage. Muscle pH was measured at 0, 2, 4, 5, 6, 8, 24, 48, and 72 h of storage by inserting the electrode of a portable meter (Testo 205; Testo) into two separate incisions of the tissue. Surface color was assessed at 0, 24, 48, and 72 h by recording CIELAB values (L*, a*, and b*) using a Minolta CR-200 colorimeter (Konica Minolta) equipped with an 8-mm aperture and a pulsed xenon lamp as the light source. Color measurements were consistently performed on the surface of the iliotibialis lateralis muscle. Two distinct locations within the same muscle were measured for each sample. When the difference between the two measurements exceeded 20%, a third location was measured, and the mean value of the measurements was calculated. To ensure adequate oxygen exposure, the samples were left uncovered for 30 min prior to color measurement.

### Evaluation of water-holding capacity

After 72 h of refrigerated storage, the entire left thigh muscle was frozen at −25°C in a household freezer. One month later, the samples were thawed overnight at 4°C. Muscle weight was measured before and after 72 h of refrigerated storage and before and after a freeze-thaw cycle. Percentage reductions were calculated and expressed as drip loss and thawing loss, respectively.

### Statistical analyses

The GSH/GSSG ratio, lipid peroxide levels, pH value, and color parameters were analyzed by two-way analysis of variance (ANOVA, factors: post-mortem time and treatment group). Other variables were analyzed by one-way ANOVA followed by Tukey’s test for multiple comparisons among groups. All analyses were performed using GraphPad Prism version 6.0 (GraphPad Software), and differences were considered statistically significant at p<0.05.

## RESULTS

### Growth performance and thigh muscle yield

Table 2 summarizes the body weight at group allocation, growth performance during the treatment period, and the body weight and thigh muscle yield at slaughter. No statistically significant differences in final body weight, average daily gain, average daily feed intake, or thigh muscle yield were observed among the control, teriflunomide, and vitamin E groups.

### Degree of oxidative stress

**Figure 1:**
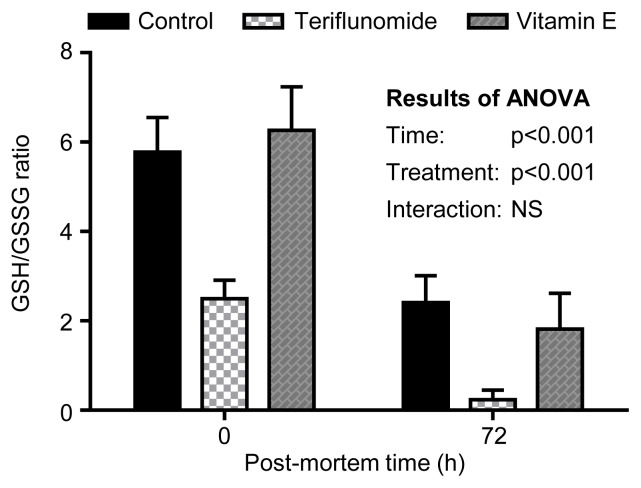
Effect of experimental treatments on glutathione content in broiler thigh muscles. The ratio of reduced glutathione (GSH) to oxidized glutathione (GSSG) was measured immediately after slaughter and after 72 h of refrigerated storage. Data are presented as means±standard errors. Two-way analysis of variance (ANOVA) with time and treatment as factors was performed, and statistical significance was set at p<0.05. NS, not significant.

**Figure 2:**
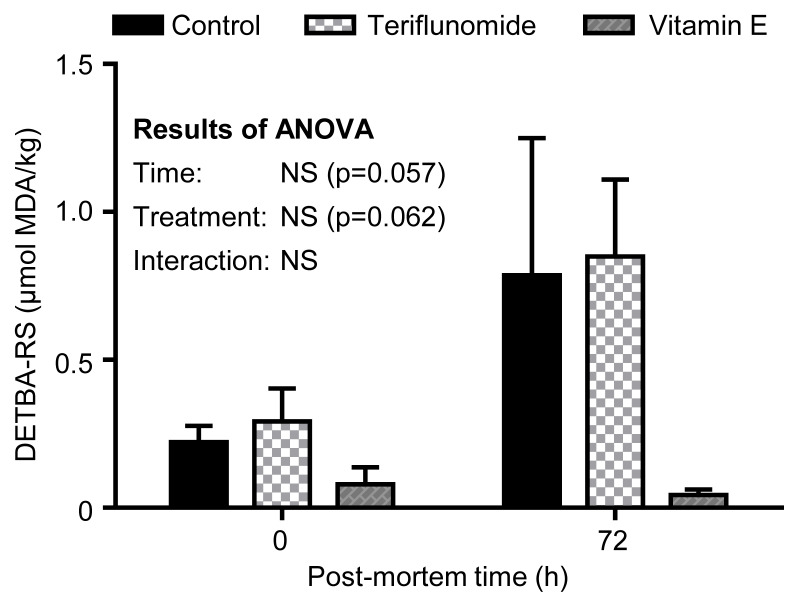
Effect of experimental treatments on lipid peroxide levels in broiler thigh muscles. The levels of 1,3-diethyl-2-thiobarbituric acid-reactive substances (DETBA-RS) in the muscles were quantified by high-performance liquid chromatography immediately after slaughter and after 72 h of refrigerated storage. Data are presented as means±standard errors. Two-way analysis of variance (ANOVA) with time and treatment as factors was performed, and statistical significance was set at p<0.05. MDA, malondialdehyde; NS, not significant.

Because the accumulation of oxidative stress on biological membranes triggers ferroptosis, its degree in each group was evaluated by measuring the GSH/GSSG ratio and the lipid peroxide levels indicated by DETBA-reactive substances (DETBA-RS). As shown in Figure 1, the GSH/GSSG ratio decreased during refrigerated storage in all groups, and the values in the teriflunomide group were markedly lower than those in the other two groups both immediately after slaughter and after 72 h of storage. No statistically significant differences were observed in DETBA-RS values among groups at any storage time point (Figure 2).

### Expression of ferroptosis-related genes

**Figure 3:**
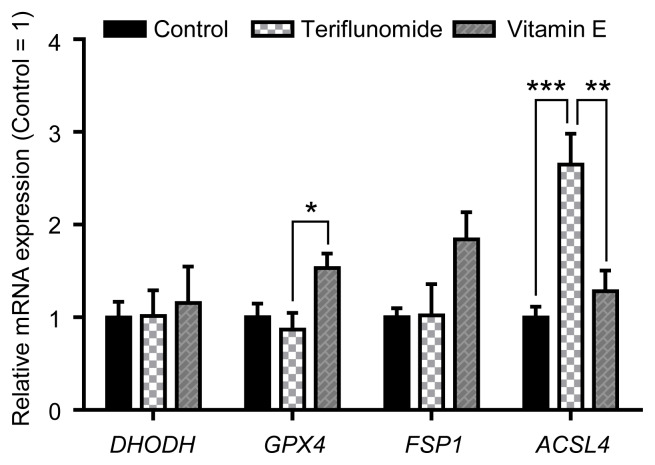
Effect of experimental treatments on the expression of ferroptosis-related genes in broiler thigh muscles. The mRNA levels of dihydroorotate dehydrogenase (*DHODH*), glutathione peroxidase 4 (*GPX4*), ferroptosis suppressor protein 1 (*FSP1*), and acyl-CoA synthetase long-chain family member 4 (*ACSL4*) were measured in thigh muscles immediately after slaughter by quantitative reverse transcription polymerase chain reaction. Data are presented as means±standard errors. One-way analysis of variance followed by Tukey’s test was performed, and one, two, and three asterisks indicate p<0.05, p<0.01, and p<0.001, respectively.

The expression of ferroptosis-related genes in thigh muscles of each group is summarized in Figure 3. Teriflunomide is a DHODH inhibitor, but it did not exert a clear effect on the gene expression of *DHODH*. Similarly, dietary supplementation with vitamin E did not cause a marked change in *DHODH* expression. The expression level of *GPX4* was slightly lower in the teriflunomide group than in the control group, whereas the vitamin E group showed higher *GPX4* expression, with a significant difference between the teriflunomide and vitamin E groups. FSP1 exerts reducing activity on biological membranes similar to that of GPX4 and DHODH. No statistically significant differences in FSP1 mRNA expression were observed among groups, although the vitamin E group showed numerically higher expression than the other groups. ACSL4 positively regulates ferroptosis by promoting the incorporation of long-chain fatty acids into biological membranes. *ACSL4* mRNA expression was significantly higher in the teriflunomide group than in the other groups.

### Meat pH, color and water-holding capacity

**Figure 4:**
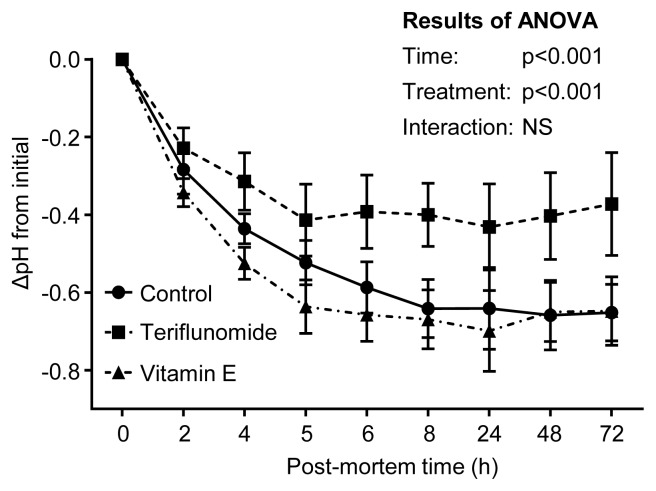
Effect of experimental treatments on the pH of broiler thigh muscles. Changes in pH during 72 h of post-mortem storage were analyzed as the difference from the initial value. Data are presented as mean±standard error. Two-way analysis of variance (ANOVA) with time and treatment as factors was performed, and p<0.05 was considered statistically significant. NS, not significant.

During 72 h of refrigerated storage, pH values showed the typical post-mortem decline in all groups (Figure 4). However, the decrease was significantly slower in the teriflunomide group than in the others. In the vitamin E group, pH was lower than that of the control group at the early post-mortem phase but eventually reached a comparable level at the end of storage.

Changes in color parameters during storage are summarized in Table 3. The L* values increased in all groups throughout the storage period, with consistently lower values in the teriflunomide group than in the control group at all time points. In contrast, the vitamin E group maintained higher L* values than the control group, and the differences between groups were statistically significant. The a* values decreased over time in all groups; however, the teriflunomide group showed higher values than the control group, whereas the vitamin E group exhibited lower values. The b* values followed a pattern similar to that of L* values, remaining lower in the teriflunomide group and higher in the vitamin E group compared with the control.

Drip loss during 72 h of refrigerated storage was negligible in all groups, and no differences were observed among the groups (data not shown). Thawing loss was numerically higher in the teriflunomide group and lower in the vitamin E group; however, these differences were not statistically significant (data not shown).

## DISCUSSION

In the present study, chickens were used as an experimental model, as they are the most widely raised livestock species worldwide and are commonly employed as avian models in basic biological research [16–19]. Thigh muscle was selected for analysis because its higher iron content, compared with breast muscle, is expected to increase susceptibility to iron-dependent lipid peroxidation, a key process in ferroptosis [20]. In one experimental group, the enzymatic activity of DHODH was pharmacologically inhibited by administering teriflunomide prior to slaughter. Teriflunomide is the active metabolite of the anti-rheumatic drug leflunomide and exerts immunosuppressive effects by inhibiting DHODH, thereby suppressing de novo pyrimidine synthesis. Recent studies have reported that DHODH inhibition also affects intracellular redox balance and lipid peroxidation, and teriflunomide has been widely used in basic research as a promoter of ferroptosis [21]. The biological activity of teriflunomide has been confirmed in chicken cells as well [22].

The other experimental group was supplemented with vitamin E before slaughter, as vitamin E is known to inhibit ferroptosis [13]. α-Tocopherol, the most biologically active form of vitamin E, was selected as a representative isoform because of its high antioxidant capacity [23]. By comparing the biochemical characteristics of muscle tissues among the control, teriflunomide-treated, and vitamin E–supplemented groups, this study aimed to gain deeper insight into the relationship between ferroptosis and meat quality.

Because ferroptosis is triggered by the accumulation of oxidative stress in biological membranes, we quantified glutathione content to assess the degree of ferroptosis. GSH functions as a cofactor for GPX4 and is converted to GSSG after the reaction. Therefore, the ratio of GSH to GSSG is considered a useful indicator of ferroptosis progression [24]. Lipid peroxide level is another representative marker of ferroptosis [25], and the amount of MDA was determined as DETBA-RS in this study. A decrease in the GSH/GSSG ratio was observed in the thigh muscle of the teriflunomide group, whereas DETBA-RS levels in the vitamin E-supplemented group were numerically lower, without reaching statistical significance. Expression analysis of ferroptosis-related genes revealed that the mRNA levels of *GPX4* and *FSP1*, both negative regulators of ferroptosis [4], were increased in the vitamin E group. Although the molecular mechanism by which vitamin E upregulates these genes remains unclear, a similar trend has been reported in other studies using chickens [26]. In contrast, expression of *ACSL4*, a key enzyme that promotes ferroptosis [4], was elevated in the teriflunomide group. Taken together, these biochemical and transcriptional findings may support the notion that ferroptosis was promoted in the teriflunomide group and suppressed in the vitamin E group.

During the post-mortem period, glycogen stored in muscle is anaerobically degraded to lactic acid. Consequently, the accumulation of lactic acid leads to a decrease in pH, which is a typical biochemical event observed in post-mortem skeletal muscle. Muscle pH is an important characteristic directly associated with the sensory and hygienic qualities of meat [27]. In the present study, pre-slaughter administration of teriflunomide markedly suppressed the post-mortem decline in the pH of broiler thigh muscle. In contrast, the pH of the vitamin E group was slightly lower than that of the control group. Although dietary vitamin E generally has little effect on meat pH, some studies have reported that it can result in a lower early post-mortem pH [28], which is consistent with our findings. In this context, several studies have demonstrated that reactive oxygen species (ROS) can inhibit the activity of glycolytic enzymes [29]. Therefore, the delayed production of lactic acid and the higher pH observed in the thigh muscle of the teriflunomide group may have resulted from ROS accumulation induced by DHODH inhibition. Similarly, the slightly lower pH in the vitamin E group might also be attributable to the reduced generation of ROS.

This study is the first to demonstrate that DHODH activity can influence meat quality traits. One particularly interesting finding was the alteration of color parameters: pre-slaughter administration of teriflunomide decreased L* and b* values while increasing a* values. Since consumers often use meat color not only as an indicator of freshness but also as a criterion for detecting abnormalities, our results highlight the significance of DHODH activity in the regulation of meat color. The biochemical properties of myoglobin, a key pigment determining meat color, are strongly affected by pH levels [30]. Therefore, the observed changes in color in the teriflunomide group are likely attributable to the corresponding alterations in muscle pH.

Although DHODH has recently attracted attention as a suppressor of ferroptosis, it has classically been recognized for its role in pyrimidine synthesis. Therefore, when evaluating the effects of teriflunomide on meat quality traits in this study, it is necessary to consider the potential involvement of both ferroptosis and pyrimidine synthesis. Notably, the changes in L*, a*, and b* values, as well as the effects on pH observed in the teriflunomide group, were comparable to those previously observed in chicken muscle under pharmacological inhibition of GPX4, the central regulator of ferroptosis [7]. Since ferroptosis is a form of cell death accompanied by membrane disruption, its promotion is thought to reduce water-holding capacity in meat [6]. In this study, although no statistically significant differences were observed among groups, the DHODH group showed numerically higher thawing loss. These findings suggest that the alterations in meat quality traits observed upon DHODH inhibition are more likely mediated by the extent of ferroptosis rather than by pyrimidine synthesis. Furthermore, considering the reductive role of DHODH at the mitochondrial membrane, our results may draw attention to the importance of mitochondria-dependent ferroptosis in determining meat quality.

Vitamin E has been widely used to supplement livestock diets to improve animal health and meat stability by reducing oxidative damage. In our study, dietary vitamin E increased L* and b* values but decreased a* values, indicating brighter, more yellow, and less red meat. Similar results have been reported by others [31,32], although the effects of vitamin E on meat color vary depending on factors such as dosage, feeding duration, and muscle type [33]. Notably, the changes observed in pH and color parameters of the thigh muscles from vitamin E-supplemented broilers were completely opposite to those observed in the teriflunomide-treated group. Considering that vitamin E supplementation and DHODH inhibition exert opposing effects on ferroptosis, these results are persuasive and further support the involvement of ferroptosis in post-mortem muscle changes.

Advances in animal genetics, breeding, and nutrition have led to the development of technologies that enable the efficient production of high-quality meat. Nevertheless, deterioration of meat quality resulting from atypical biochemical alterations in muscle tissue before or after slaughter is still occasionally observed across livestock species. These abnormalities include the well-characterized meat quality defects known as dark, firm, and dry (DFD) and pale, soft, and exudative (PSE) meat [34]. DFD meat typically arises from insufficient glycogen reserves prior to slaughter, which cause a high ultimate pH and a dark appearance. In contrast, PSE meat results from an excessively rapid post-mortem pH decline while the muscle temperature remains high, producing a pale color.

The present study demonstrated that teriflunomide administration increased muscle pH while decreasing the L* values, suggesting that reduced DHODH activity may influence the development of PSE-like changes in skeletal muscle through ferroptosis-associated post-mortem metabolic alterations. One of the major factors contributing to PSE development is excessive accumulation of lactate [34]. Given that the activities of enzymes involved in lactate production from glycogen can be suppressed by ROS [29], the higher pH observed in the teriflunomide group may be attributable to ROS-mediated inhibition of lactate accumulation. However, ROS generation and post-mortem oxidative stress are generally regarded as factors that deteriorate meat quality, and the changes observed here should not be interpreted as universally beneficial. Rather, the present findings indicate that any attenuation of PSE-like characteristics may occur at the expense of an increased risk of DFD-like properties, reflecting a potential trade-off between these two types of meat quality defects. In contrast, dietary supplementation with vitamin E resulted in meat quality characteristics opposite to those observed in the teriflunomide group. Because vitamin E supplementation can be readily applied in practical animal production, these findings may contribute to the development of vitamin E–based strategies aimed at modulating post-mortem oxidative processes by regulating ferroptosis-related pathways, thereby helping to control meat quality. Nevertheless, the relationships discussed above remain speculative, and further studies are required to directly verify the interplay among ferroptosis, glycolytic activity, and post-mortem meat quality traits.

Ferroptosis-related lipid peroxidation occurs in post-mortem muscle, accompanied by the accumulation of lipid radicals and a decrease in GPX4 activity [35]. Such oxidative damage has been associated with reduced water-holding capacity and changes in color and texture [35,36], implying that ferroptosis could be involved in the regulatory mechanisms underlying meat quality. The present results support this notion by showing that muscle pH was higher and color traits were altered in post-mortem muscle following acceleration of ferroptosis. Further careful investigation is required to fully elucidate the role of ferroptosis in post-mortem muscle because cell death is tightly regulated by multiple pathways, and ferroptosis is known to function in concert with other forms of cell death [37]. To address this issue, ultrastructural analysis using electron microscopy would be a valuable approach for future studies. In addition, although the present study examined the expression of ferroptosis-related genes at the mRNA level, analysis at the protein level would provide further insights. In this study, 21-day-old broiler chickens were used for practical reasons to reduce the amount of teriflunomide required. However, because commercial broilers are typically slaughtered at an older age, future studies using birds over one month of age are needed to assess the applicability of the present findings to commercial production. Another limitation is that sensory evaluation could not be performed by human panelists, as the thigh meat contained teriflunomide. Furthermore, our previous research demonstrated that the expression of ferroptosis-related genes varies markedly among muscle types [38], suggesting that the role of ferroptosis should also be investigated in relation to species differences and muscle fiber composition.

## CONCLUSION

In conclusion, this study demonstrated that inhibition of DHODH, a key regulator of ferroptosis, via pre-slaughter administration of teriflunomide in broilers led to increased post-mortem oxidative stress in thigh muscles, accompanied by alterations in meat quality traits, including elevated pH, decreased L* values, and increased a* values. In contrast, the group receiving vitamin E, which is expected to suppress ferroptosis, exhibited meat quality characteristics that were completely opposite to those observed in the teriflunomide group. These findings highlight the significance of ferroptosis in the post-mortem biochemistry of skeletal muscle and provide valuable insight into the role of ferroptosis in the regulation of meat quality.

## Figures and Tables

**Table 1:** Primer sequences used for quantitative reverse transcription-polymerase chain reaction

mRNA	Forward primer	Reverse primer
*DHODH*	GAAGTTGGAACTGTCACGCC	CTTGCCCAGGTTGACTCCAA
*GPX4*	CTTCGTCTGCATCATCACCAA	TCGACGAGCTGAGTGTAATTCAC
*FSP1*	TCATTGACATGGAAAGCGCC	CCACTACTAGGATGCGCTCA
*ACSL4*	ATCACCAGTGCAGAGCTTCT	AGCTCTTCCACTGTCTGCAT
*GAPDH*	CTGGAGAAACCAGCCAAGTA	ATGGCTGTCACCATTGAAGT

*DHODH*, dihydroorotate dehydrogenase; *GPX4*, glutathione peroxidase 4; *FSP1*, ferroptosis suppressor protein 1; *ACSL4*, acyl-CoA synthetase long-chain family member 4; *GAPDH*, glyceraldehyde-3-phosphate dehydrogenase.

**Table 2:** Growth performance and thigh muscle yield in broiler chickens

	Control	Teriflunomide	Vitamin E	p level
Body weight at group allocation (g)	546±10	523±18	535±29	NS
Body weight at slaughter (g)	830±17	773±25	810±41	NS
Average daily gain (g/day)	40.5±1.2	35.7±2.0	39.3±2.0	NS
Average daily feed intake (g/day)	61.0±0.5	59.5±1.1	61.7±0.8	NS
Thigh muscle weight (g)	162.8±6.5	175.0±8.1	167.5±4.6	NS

Data were analyzed by a one-way analysis of variance and are presented as means±standard error.

NS, not significant.

**Table 3:** Effects of experimental treatments on color parameters of broiler thigh muscle

Treatment		Post-mortem time				Results of ANOVA		
	
		0 h	24 h	48 h	72 h	Time	Treatment	Interaction
L*	Control	50.68±0.95	52.71±0.78	52.66±0.75	52.95±0.61	p<0.01	p<0.01	NS
	Teriflunomide	48.40±1.47	50.34±1.41	51.90±1.25	52.15±1.08			
	Vitamin E	52.02±0.67	53.15±0.76	54.03±0.74	54.10±0.86			
a*	Control	4.07±0.37	3.10±0.52	3.34±0.52	3.51±0.46	NS	p<0.001	NS
	Teriflunomide	5.50±0.79	4.19±0.82	4.31±0.62	4.66±0.77			
	Vitamin E	3.81±0.60	2.79±0.74	2.81±0.57	2.94±0.56			
b*	Control	6.17±0.48	3.97±0.80	3.47±0.66	3.16±0.27	p<0.001	p<0.05	NS
	Teriflunomide	5.12±0.76	3.45±0.57	3.21±0.56	3.27±0.55			
	Vitamin E	6.75±0.81	4.84±0.64	4.64±0.90	4.16±1.12			

Data were analyzed by a two-way analysis of variance (ANOVA) and are presented as means±standard error.

NS, not significant.

## Data Availability

Upon reasonable request, the datasets of this study can be available from the corresponding author.
